# Resource-optimized cnns for real-time rice disease detection with ARM cortex-M microprocessors

**DOI:** 10.1186/s13007-024-01280-6

**Published:** 2024-10-16

**Authors:** Hermawan Nugroho, Jing Xan Chew, Sivaraman Eswaran, Fei Siang Tay

**Affiliations:** 1 Electrical and Electronic Engineering Department, University of Nottingham Malaysia, Jln Broga, 43500 Semenyih, Malaysia; 2 Department of Electrical and Computer Engineering, Curtin University Malaysia, 98009 Miri, Malaysia; 3 Faculty of Engineering, Computing and Science, Swinburne University of Technology Sarawak Campus, Q5B, 93350 Kuching, Malaysia

**Keywords:** ARM Cortex-M microprocessors, Convolutional neural networks, Rice disease detection, Agricultural productivity

## Abstract

This study explores the application of Artificial Intelligence (AI), specifically Convolutional Neural Networks (CNNs), for detecting rice plant diseases using ARM Cortex-M microprocessors. Given the significant role of rice as a staple food, particularly in Malaysia where the rice self-sufficiency ratio dropped from 65.2% in 2021 to 62.6% in 2022, there is a pressing need for advanced disease detection methods to enhance agricultural productivity and sustainability. The research utilizes two extensive datasets for model training and validation: the first dataset includes 5932 images across four rice disease classes, and the second comprises 10,407 images across ten classes. These datasets facilitate comprehensive disease detection analysis, leveraging MobileNetV2 and FD-MobileNet models optimized for the ARM Cortex-M4 microprocessor. The performance of these models is rigorously evaluated in terms of accuracy and computational efficiency. MobileNetV2, for instance, demonstrates a high accuracy rate of 97.5%, significantly outperforming FD-MobileNet, especially in detecting complex disease patterns such as tungro with a 93% accuracy rate. Despite FD-MobileNet’s lower resource consumption, its accuracy is limited to 90% across varied testing conditions. Resource optimization strategies highlight that even slight adjustments, such as a 0.5% reduction in RAM usage and a 1.14% decrease in flash memory, can result in a notable 9% increase in validation accuracy. This underscores the critical balance between computational resource management and model performance, particularly in resource-constrained settings like those provided by microcontrollers. In summary, the deployment of CNNs on microcontrollers presents a viable solution for real-time, on-site plant disease detection, demonstrating potential improvements in detection accuracy and operational efficiency. This study advances the field of smart agriculture by integrating cutting-edge AI with practical agricultural needs, aiming to address the challenges of food security in vulnerable regions.

## Introduction

All over the world, rice is the primary staple food for a large percentage of people, which means its production process is the backbone for ensuring the food security of many states. In Malaysia, the agricultural sector is faced with the dilemma of keeping up, if not exceeding, the domestic rice sufficiency together with various risk factors. Recent statistics from the Department of Statistics Malaysia highlighted a concerning trend: the rice self-sufficient ratio went from 65.2% recorded in 2021 to 62.6% in 2022 [7]. The reduction, consequently, shows rice production’s susceptibility to several factors, like plant diseases and the environmental hazards that beset some country’s northern state, which among others, contribute to 60% of the country’s rice output (Omar, et al. [19]). The rice production that is mainly concentrated in these areas increases the vulnerability of the agricultural sector to one of the most damaging consequences of the emergence of plant diseases as well as unfavorable weather conditions.

Early detection of those potentially infected plants is crucial to avoid the spread of diseases in plantations which can have very high costs in terms of time and financial resources (Fabrizio, et al. [5]). However, complex procedures for plant disease identification leading to difficulties for agronomists in distinguishing diseases especially with multiple symptoms (Wei, Rizab and Nugrohoa [24]). Manual disease identification is inefficient, often resulting in misdiagnoses and ineffective treatments (Dokic, Blaskovic and Mandusic [9]).

Considering the challenges, there has been a growing interest in harnessing smart agricultural technologies to mitigate risks and enhance productivity. Among these technologies, artificial intelligence (AI), particularly Convolutional Neural Networks (CNNs), stands out as having the greatest potential to revolutionize plant disease detection and management. CNNs, a class of deep neural networks, are widely recognized for their exceptional performance in image recognition and classification. In the context of agriculture, CNNs have demonstrated remarkable accuracy in identifying various plant diseases by analyzing leaf images. This capability facilitates early disease detection, which is crucial for preventing further disease spread and minimizing crop losses. However, it’s worth noting that existing deep learning applications in agriculture often prioritize development over deployment, with significant emphasis on ensuring the robustness of plant disease detection models at the application layer. Innovations such as edge devices for sunlight adjustment and AI for plant disease detection have been patented separately [10, 16, 25], but their combined use remains largely unexplored.

Next challenge is that most deep learning models are deployed on cloud servers, necessitating stable and uninterrupted internet access. This reliance poses challenges in rural or remote farming areas where such connectivity is limited, as seen with deep learning models used for tea leaf disease detection on a Platform-as-a-Service (PaaS) cloud, accessed via smartphones [14]. These dependencies create significant barriers in settings lacking robust internet infrastructure.

Lastly, traditional deep learning deployments rely on centralized servers with substantial computational power, which may not be practical or cost-effective for agricultural uses. Highlighted issues include the extensive resource demands of these servers, which are often incompatible with the constraints of agricultural applications [1]. Therefore, there is an increasing demand for machine learning solutions that operate directly on edge devices, independent of centralized servers and continuous internet access. Edge Computing with AI technologies, especially on low-power, embedded devices like ARM-M based microcontrollers, offering intelligent, localized solutions that preserve privacy, reduce latency, provide real-time performance, and optimize resources (Li, et al. [15]).

The project focuses on developing an AI-driven plant disease detection model deployed on an ARM-M based microcontroller, enabling real-time monitoring of rice plant diseases while addressing the practical limitations of embedded devices. This research makes significant contributions by optimizing and deploying CNN models on resource-constrained ARM Cortex-M microprocessors. By utilizing advanced techniques such as model quantization and compression, the study strikes a crucial balance between computational efficiency and model accuracy, making it suitable for real-time application in resource-limited agricultural environments. This work will not only demonstrate enhanced disease detection accuracy in the field but also advance the integration of edge AI in agriculture, providing a scalable and localized solution that supports sustainable farming practices, particularly in remote and resource-limited areas.

## Related works

In agriculture, the application of these advanced neural networks, especially Convolutional Neural Networks (CNNs) and deep learning techniques, has revolutionized the identification and classification of diseases in crops such as rice and paddy plants. In (Sethy, et al. [23]), a comprehensive study was conducted on the identification of rice leaf diseases using machine learning and deep learning techniques. The study centres around the analysis of rice leaves images affected by four common diseases: bacterial blight, blast, brown spot, and tungro. The research assesses the performance of eleven Convolutional Neural Network (CNN) models using techniques such as transfer learning and deep feature extraction, combined with Support Vector Machine (SVM) for disease classification. The findings show that deep features extracted from the fc6 layer of CNN model types such as AlexNet, VGG16, and VGG19 exhibit superior performance when paired with SVM classifiers, as compared to the fc7 and fc8 layers. Notably, the combination of ResNet50 with SVM achieves the highest performance, boasting an impressive F1 score of 0.9838, underscoring its accuracy in disease identification. Additionally, the study evaluates the efficacy of smaller-scale models like MobileNetV2 and ShuffleNet. Remarkably, when compared to the larger ResNet50 model, MobileNetV2 combined with SVM demonstrate similar performance in terms of both accuracy and efficiency. Kaur et al. [13] presents a hybrid Convolutional Neural Network (CNN) model for the detection and classification of grapevine leaf diseases, utilizing EfficientNet with transfer learning and feature reduction techniques. Focusing on four key grape diseases—leaf blight, black rot, black measles, and stable—the model was trained on the PlantVillage dataset, achieving an accuracy of 98.7% after 92 epochs. The study emphasizes the importance of early and accurate disease detection in agriculture and demonstrates the model’s effectiveness in resource-constrained environments. The proposed framework offers practical applications for real-time disease detection in farming, enhancing crop management and sustainability.

In (Amritha, Jeena and Ebin [2]), Amritha Haridasan et al developed a deep learning system using neural networks to detect and classify diseases in paddy plants. This system achieved a validation accuracy of 91.45% and addressed the five most common paddy diseases using image processing and deep learning techniques, including ReLU and SoftMax functions for disease recognition and treatment recommendations. The methodology incorporated image acquisition, segmentation, and feature extraction techniques such as Otsu’s thresholding, Sobel edge detection, and K-means clustering, followed by disease classification through Support Vector Machine (SVM) and Convolutional Neural Network (CNN) models. The study demonstrated the superiority of CNNs over SVMs and reviewed activation and loss functions within CNNs to enhance accuracy. It also suggested possibilities for advancing the speed of disease detection by using more sophisticated deep learning approaches and expanding them across various agricultural applications.

As the accuracy of the models increases, so does their size. This trend towards larger models introduces challenges, particularly when deploying these technologies in real-world scenarios. Microcontrollers, commonly used in agricultural technology tools for their low cost, energy efficiency, and compact size, have limited computational resources. This makes it difficult to run large neural network models effectively. The practicality of implementing these advanced and sizable neural networks on microcontrollers is a major concern, requiring a careful balance between model complexity and practical applicability. This has prompted researchers and developers to focus on model optimization and compression techniques to mitigate these issues.

In [3], a method is proposed to optimize deep learning models for sound event detection (SED) on platforms with low power and low complexity. This method employs a two-stage student–teacher technique to compress advanced neural networks to a size suitable for microcontrollers, specifically targeting the ARM Cortex M4 microprocessor. The technique includes quantization and knowledge determinization, achieving a high compression ratio with minimal loss of accuracy. The research demonstrates that these optimized models perform satisfactorily, with short inference times and low power consumption, enabling the deployment of complex machine learning models on microcontrollers.

A study (De Vita, et al. [5]) conducted by the University of Messina, Missouri University of Science and Technology, and STMicroelectronics introduces an innovative approach to plant disease detection by deploying deep neural networks on STM32 microcontrollers. This method utilizes dynamic K-means compression to reduce the size and increase the efficiency of complex neural network models, facilitating their use in agricultural applications. The technique ensures highly accurate disease detection and addresses the challenges of implementing advanced AI technologies on low-power, resource-constrained devices, marking significant progress in smart agriculture.

A collaborative effort by the University of Messina, Missouri University of Science and Technology, and STMicroelectronics (de Vita, et al. [6]), demonstrates the effectiveness of Quantized Convolutional Neural Networks (Q-CNNs) in diagnosing diseases in coffee plants. Utilizing the latest STM32 microcontroller family, this research highlights how a smart farming application can achieve a 96% accuracy in disease identification. The study primarily explores the use of AI and edge computing in agricultural technology, applying compaction and quantization techniques to adapt neural network models for environments with limited power and resources.

These recent studies collectively highlight the transformative role of artificial intelligence (AI) and machine learning in agriculture, leading to more efficient, accurate, and accessible methods for detecting plant diseases. By utilizing advanced neural networks and innovative compression techniques, researchers are addressing the computational resource limitations previously encountered, thereby setting new standards for smart agriculture. These developments not only improve our understanding of plant diseases but also pave the way for sustainable farming practices, representing a significant advancement in agricultural technology.

## Methodology

### Dataset for the development of the project

**Fig. 1:**
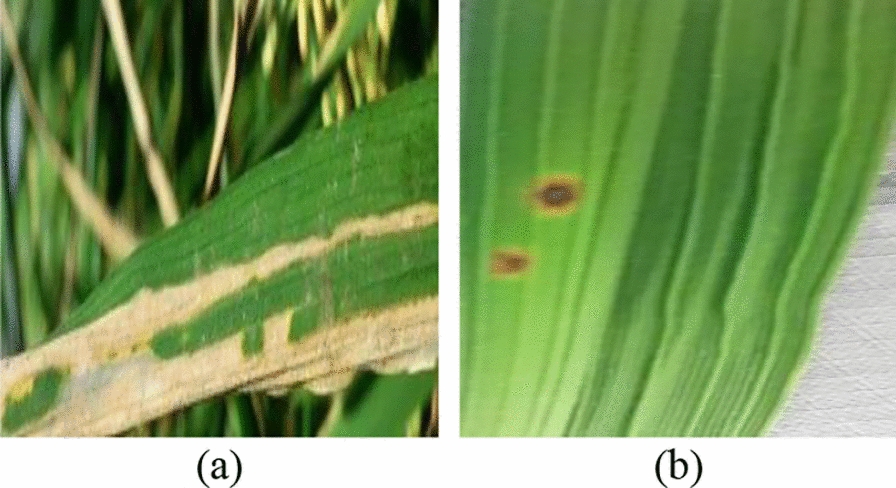
**a** Bacterial Blight and **b** Brown Spot from Dataset 1

**Fig. 2:**
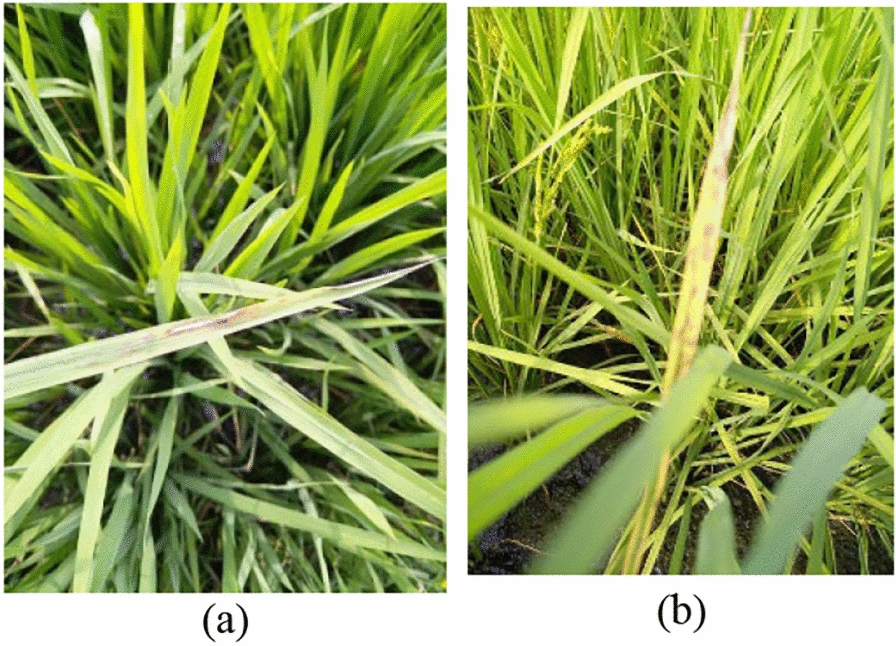
**a** Bacterial Blight and **b** Brown Spot from Dataset 2

The project employs two datasets. Dataset 1, curated by Sethy et al. (Sethy, et al. [23]), consists of 5932 images across four distinct classes of rice leaf diseases, specifically captured to highlight the clarity of disease manifestations. Although these images are of high quality, the dataset size is relatively small. Conversely, Dataset 2, compiled by Doctor and Petchiammal (Doctor, (Samy) and Petchiammal [8]), includes 10,407 images covering nine classes of rice leaf diseases and one class for healthy leaves. The images in Dataset 2 are of slightly lower quality; they typically capture the entire leaf, requiring detailed examination to pinpoint disease areas. Initially, Dataset 1, consisting of high-quality images with clear disease manifestations, is used for foundational model training. This ensures the model has a strong basis for detecting key disease patterns. However, to prevent any bias stemming from reliance on a smaller, more controlled dataset, we then introduce Dataset 2, which contains additional classes and more diverse image quality. The incremental integration of the six unique classes from Dataset 2 serves to expand the model’s scope, allowing it to handle real-world variations and complexities in disease detection. Both datasets are utilized in this study to facilitate comprehensive disease detection analysis. The use of these two datasets allows the model to benefit from both high-quality, clearly labeled disease examples (Dataset 1) and a more diverse, real-world representation of diseased and healthy leaves (Dataset 2). This combination is essential for training a robust model that can generalize well to different environments and varying image qualities, ensuring the system can accurately identify diseases in a variety of field conditions. Samples of images from both datasets are presented in the following figures. Figure 1 shows examples of Dataset 1 and Fig. 2 shows examples of Dataset 2. The details of Dataset 1 and Dataset 2 are shown in Tables 1 and 2 respectively.

**Table 1:** Details of dataset 1

Leaf disease	Number of images
Bacterial blight	1584
Blast	1440
Brown spot	1600
Tungro	1308
Total	5932

**Table 2:** Details of dataset 2

Leaf disease	Number of images
Bacterial blight	479
Bacterial leaf streak	380
Bacterial panicle blight	337
Dead heart	1442
Downy mildew	620
Hispa	1594
Normal	1764
Blast	1738
Brown spot	965
Tungro	1088
Total	10,407

The initial model development will be based on Dataset 1, with fine-tuning and model extension utilizing Dataset 2. Given the lower image quality in Dataset 2 and the redundancy of the four disease classes already included in Dataset 1, these overlapping images will be excluded to prevent any potential compromise to the model’s accuracy or performance. Instead, the six additional unique classes from Dataset 2 will be incrementally integrated into the model. The ‘normal’ class will be introduced first, as it is crucial for training the model to effectively identify healthy paddy plants, thereby minimizing false positives. By systematically incorporating each new class, the model’s performance can be closely monitored and optimized, ensuring that every addition enhances both the model’s accuracy and overall effectiveness.

### Model development and quantization

The project focuses on models optimized for deployment on STM32 platforms capable of running artificial intelligence (AI) tasks, such as the STM32H747i board, as recommended by STM. Five models are considered: FD-MobileNet, MobileNet V1, MobileNet V2, ResNet V1, and SqueezeNet V1.1. These models are not only readily available but also highly customizable to suit specific application needs, making them ideal for deployment in resource-constrained environments such as STM32 series.

In the development process, standard image augmentation techniques were applied to a deliberately chosen imbalanced dataset. This strategy was employed to enable a direct comparison with the results of the referenced study. Using an imbalanced dataset can strategically enhance model performance for specific tasks under certain conditions. This is especially pertinent in agricultural applications where the early detection of severe rice plant diseases is critical, outweighing the need for uniform accuracy across all disease classes. Some diseases pose a greater threat than others, making their early identification essential to prevent extensive damage [18]. The system configuration used for training the data in this study includes a 6 GB RTX GEFORCE 3060 GPU, which provides significant computational power. Additionally, the training process is supported by a 12th Gen Intel i9 CPU, ensuring efficient processing and rapid execution of the deep learning tasks.

Among the models tested, MobileNets were found to be more efficient and accurate than ResNets and SqueezeNets. Notably, FD_MobileNet, despite being more resource efficient than MobileNet V2, showed lower accuracy, underscoring the need to balance computational resource consumption with model accuracy. Both models are from the same family, which simplifies the development process due to their common architectural principles.

The primary distinction between FD_MobileNet and MobileNetV2 lies in their approach to downsampling. FD_MobileNet uses a faster downsampling method early in the training process, which is the main differentiator between the two models. Despite this, the models share a similar development framework, which aids in comparative analysis. This focus allows for an in-depth evaluation of how minor architectural changes can influence performance metrics and computational efficiency, thereby guiding decisions on the trade-off between resource use and accuracy.

**Fig. 3:**
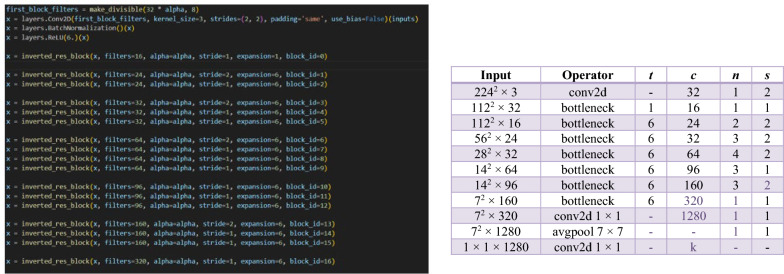
MobileNetV2 Architecture

In the MobileNetV2 architecture as shown in Fig. 3 (Sandler, et al. [20]), the width multiplier, also known as *alpha*, plays a vital role by dynamically scaling the network’s width to adjust the computational load. The architecture begins with a standard 2D convolutional layer with 32 output channels, setting the stage for the feature extraction process. Following this are the inverted residual blocks, central to MobileNetV2’s efficiency and effectiveness. Each block uses an expansion factor, denoted as *t*, which temporarily increases the number of input channels, enhancing the network’s ability to process features. The number of output channels per block is specified by *c*, and the repeat factor, *n,* determines how many times blocks with the same configuration are used consecutively within the network. The stride, s, affects the output’s spatial dimensions, with a stride of two halving the dimensions of the feature map. Notably, the first inverted residual block is unique, used only once (*n* equals 1) and has fewer output channels [16]. The architecture concludes with a global average pooling layer, followed by a dropout layer (if specified), and a fully connected layer that employs a SoftMax activation function for multi-class tasks or a sigmoid function for binary classification. The model is constructed in Keras with defined inputs and outputs, and the model’s configuration is named based on the alpha value, allowing for different model widths. This setup enables the creation of various versions tailored to specific computational resources or application needs.

**Fig. 4:**
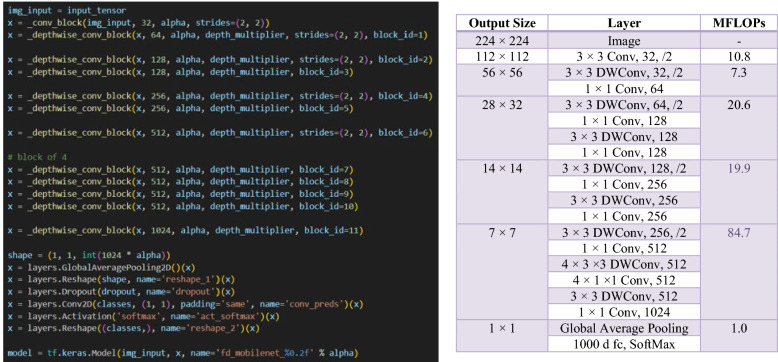
FD_MobileNet Architecture

The FD-MobileNet model construction starts with an input tensor, followed by a sequence of depth wise convolution blocks, each uniquely parameterized as shown in Fig. 4. A global average pooling layer then condenses each feature map to a single value, effectively reducing dimensionality and preparing the data for final classification. The model concludes with a reshape layer, a dropout layer for regularization, and a final Conv2D layer with a SoftMax activation function tailored to the desired number of output classes. The model is compiled into a TensorFlow Keras Model, ready for training and application, with the naming convention reflecting the adjustable width parameter, *alpha*.

For deployment on devices with limited resources, such as the STM32H747I, reducing the model’s size while maintaining performance is essential. This is achieved through post-training quantization using the TensorFlow Lite Converter, which transforms the Keras model from the larger *h5* format to the more compact and efficient *tflite* format. The *tflite* model not only significantly reduces size but also enhances inference speed, making it optimal for edge devices. During quantization, input samples reflecting real-world data are generated, and the model’s data types are converted to int8 or uint8 to further minimize size. These optimizations ensure efficient runtime performance on resource-constrained hardware.

### Model deployment

**Fig. 5:**
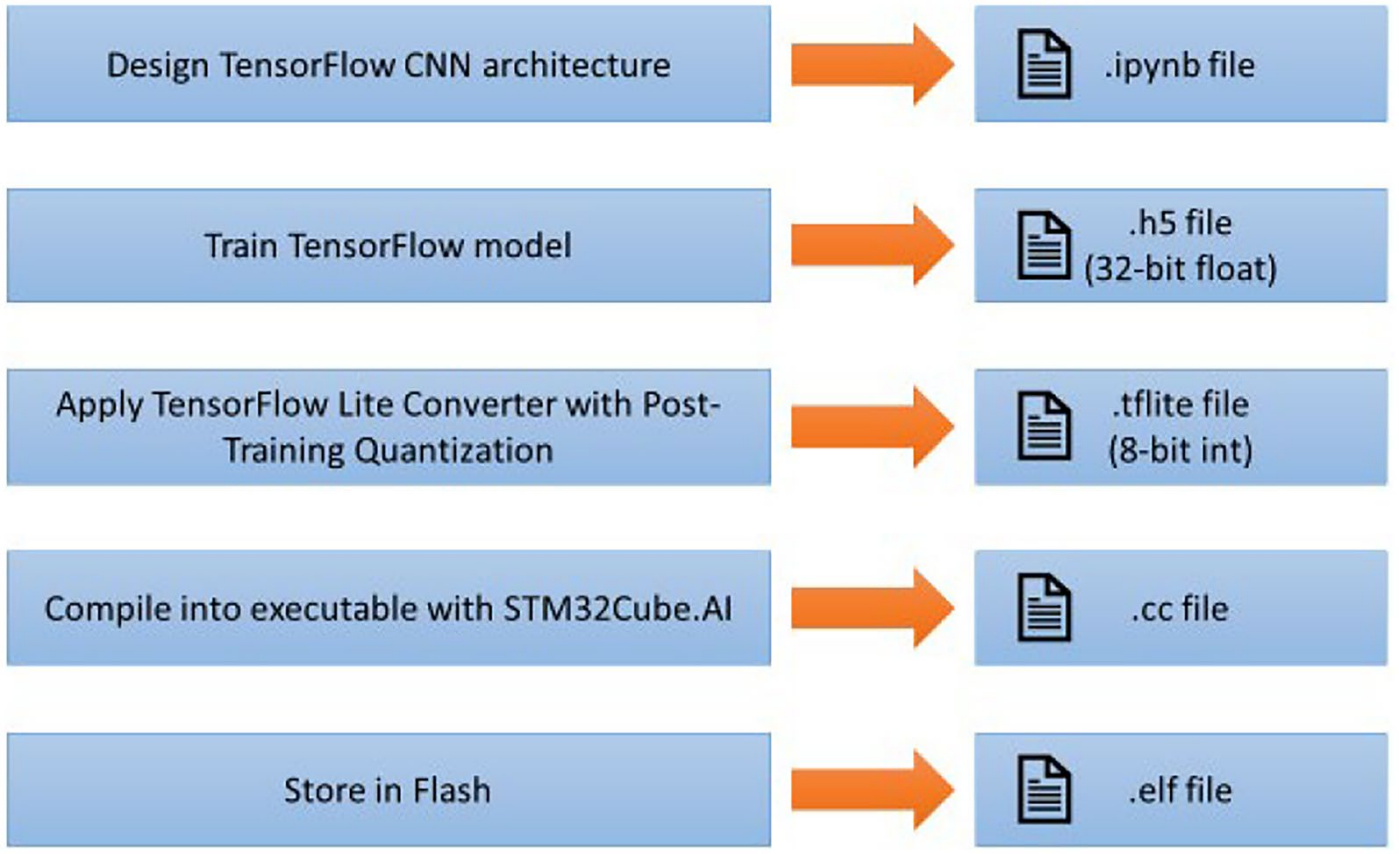
Conversion and deployment TensorFlow model to STM32 (de Vita, et al. [6])

To deploy a trained TensorFlow model on STM32 microcontrollers, it must first be converted into a suitable format. This conversion process is facilitated by STM32Cube. AI tools, which can transform a *tflite* model into a *cc* source file, the latter being appropriate for embedding into microcontroller-based applications. Figure 5 shows the overall deployment process.

**Fig. 6:**
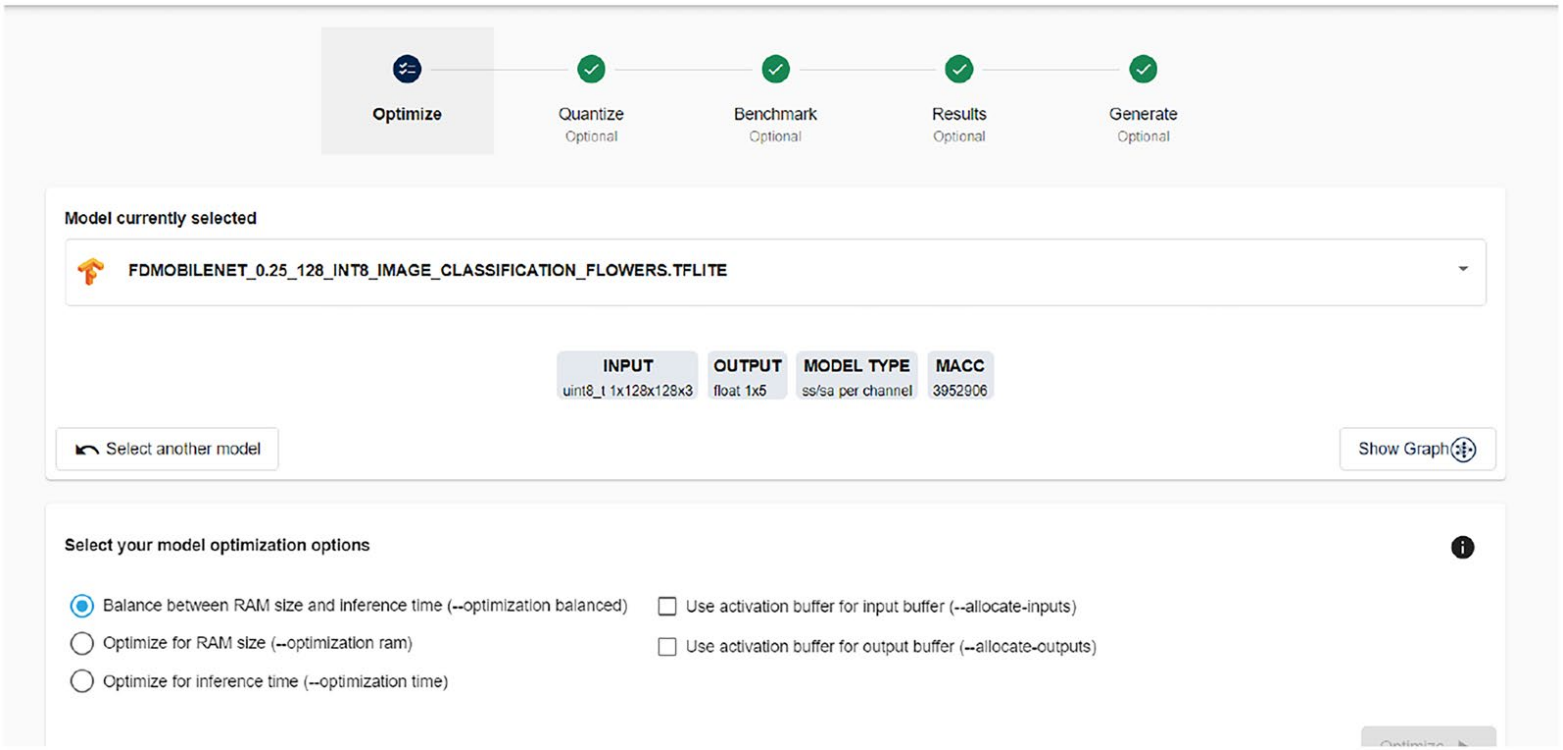
Point of view of cloud developer for deployment

The STM32Cube X-Cube-AI package is a utility that integrates directly into STM32CubeIDE. It is typically executed via command prompt, allowing for the conversion of AI models without a graphical interface. Another approach is using the STM32Cube.AI Developer Cloud. The STM32Cube.AI Developer Cloud offers a user-friendly, visual interface for managing AI model conversion. Figure 6 shows the cloud developer’s point of view.

**Fig. 7:**
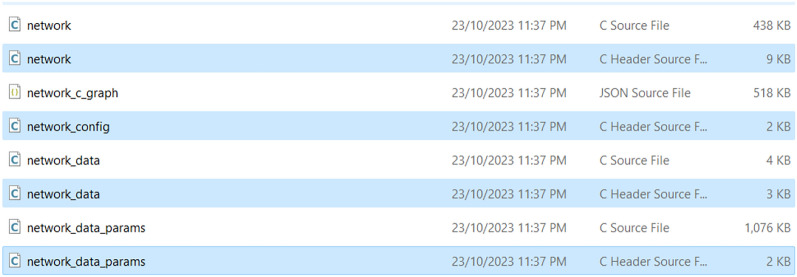
X-Cube-AI generated C files

The diagram illustrates a workflow enabling users to execute steps for deploying a TensorFlow model on an STM32 board via the STM32Cube.AI platform. It supports the entire lifecycle of AI model deployment, including creation, optimization, benchmarking, and code generation. The cloud platform provides tools to optimize the model. Users can choose to keep default settings. This step refines the model to ensure it runs efficiently on the target microcontroller. Next, quantization reduces the precision of the model’s numerical data, which can significantly decrease the model size and increase inferencing speed without substantially impacting accuracy. For benchmarking, the step involves selecting an STM32 board model and benchmarking the model to assess its RAM and Flash memory usage. Benchmarking is vital to ensure the chosen board can support the model’s memory and computational requirements. Finally, the platform generates optimized C code for the pre-trained model as shown in Fig. 7. Users must select the appropriate board model to tailor the code correctly. After a brief processing period, a zip file containing the.C and.H files along with the necessary STM32Cube.AI library is provided for download.

For integration into STM32CubeIDE, the generated C files, which include network.c and network.h, are integrated into the STM32CubeIDE. These files encapsulate the neural network architecture, detailing each layer and its parameters, while the learned parameters (weights and biases) are stored within network_data.c and network_data.h. The FP-AI-Vision package, utilized for this integration, supplies a pre-configured STM32CubeIDE project that includes all necessary libraries and modules to support computer vision applications on the STM32H747i-Disco board.

## Results

### Model development – computer simulation and hardware verification results

To evaluate the computer resources used by a model, it is important to consider the Multiply-Accumulate Operations (MACCs). A model with a high number of MACCs indicates extensive operations for data processing, which suggests increased usage of hardware resources. These operations are common in data processing and machine learning but can burden hardware (Schober, Najafi and TaheriNejad [22]). Flash weight optimization is critical in efficiently managing memory usage during model inference by optimizing how weights are encoded. Models with high demands for flash weight might face memory management challenges (Han, Mao and Dally [11]). Additionally, RAM Activation involves the use of Random Access Memory (RAM) in computing systems, where significant activation can strain memory resources (Chen, et al. [4]). Inference time measures the speed at which a model provides predictions or responses after input. Ideally, models should aim for minimal inference time to improve efficiency and responsiveness.

Experiment 1 was conducted to ascertain the optimal model for a plant disease detection application. In this experiment, standard image augmentation techniques were applied, and an imbalanced dataset was deliberately chosen, as outlined in reference paper [25]. This approach allows for direct comparison with the referenced study and is strategic in enhancing model performance for specific tasks. In cases like agricultural applications, where early detection of certain virulent rice plant diseases is critical, an imbalanced dataset can prioritize the identification of more severe diseases, even if it doesn’t ensure uniform accuracy across all classes. This prioritization helps address diseases that pose a greater threat, enabling early intervention to prevent widespread damage. Table 3 shows the results of Experiment 1, highlighting FD_MobileNet as the most resource-efficient model. It requires fewer Multiply-Accumulate (MACC) operations, achieves faster inference times, and maintains a strong accuracy rate of 98.44%, making it ideal for resource-constrained environments. In contrast, SqueezeNet achieves the highest accuracy, but its substantial model size presents deployment complexities on the STM32H747i-Disco board. MobileNetV2, ranking second in accuracy, demonstrates efficient utilization of computational resources. Given that both MobileNetV2 and FD_MobileNet stem from the same MobileNet architecture, their development processes are anticipated to exhibit similarities. Therefore, subsequent experiments will focus on comparing and contrasting FD_MobileNet and MobileNetV2.

**Table 3:** Experiment 1: evaluation of the 5 state-of-the-art models

Model type	MACCs (M)	Ram activations (KiB)	Flash weights (KiB)	Inference time (ms)	Quantized model accuracy, %
FD_mobilenet 0.25	3.96	67.46	197.15	19.81	98.44
Mobilenet_v2_0.35	19.10	262.22	543.41	100.91	98.64
Resnet_v1_depth_8	200.64	546.05	138.19	580.98	96.78
SqueezeNet_v1.1	81.21	262.18	803.34	261.25	99.22
Mobilenet_v1_alpha_0.25	13.61	88.87	300.44	53.93	97.47

To enhance a model’s performance, fine-tuning its hyperparameters is essential. These hyperparameters include the number of epochs, solver type, learning rate policy, and dropout rate. In this experiment, two solvers were evaluated: Stochastic Gradient Descent (SGD) and Adam (Adaptive Moment Estimation). SGD adjusts model parameters effectively by using the gradient of the loss function and is suitable for edge devices due to its lower computational demands. Conversely, Adam enhances optimization by maintaining a moving average of both the gradients and squared gradients, allowing for adaptive learning rate adjustments based on past gradient data (Park [20]).

For learning rate policies, the options tested were “step” and “reduce on plateau.” The “step” policy reduces the learning rate by a factor of ten every 50 epochs, while the “reduce on plateau” policy lowers the learning rate when improvements plateau, aiding in model convergence during periods of minimal progress. This approach adjusts the learning rate based on the model's performance over time.

Dropout, a technique used to prevent overfitting in neural network training, involves randomly disabling a set percentage of neurons during each training phase. In this experiment, dropout rates of 0.2, 0.5, and 0.8 were tested, with a 0.2 rate indicating that 20% of neurons are deactivated in each training iteration, promoting the model's ability to learn generalized features.

The initial training phase utilized a 50-epoch configuration, with the Adam optimizer proving more effective, yielding higher accuracies (Sethy, et al. [23]). Training was later extended to 150 epochs. Unlike prior studies that used transfer learning, this approach was avoided to maintain a manageable model size for deployment on STM32 boards.

Table 4 indicated that Model 8 achieved the highest accuracy at 99%. At 50 epochs, configurations employing the “step” learning rate policy performed better than those using “reduce on plateau.” However, this trend reversed over an extended 150 epochs, with “reduce on plateau” showing greater effectiveness due to its dynamic learning rate adjustments. Ultimately, the experiment achieved a quantized accuracy of 99% using the Adam optimizer, a higher dropout rate of 0.8, and the “reduce on plateau” learning rate policy across 150 epochs. This combination of Adam, increased dropout, and adaptive learning rate adjustments proved highly effective for the FD_MobileNet structure, optimizing performance for deployment on resource-limited hardware such as the STM32 board.

**Table 4:** Experiment 2 parameter selection for FD_MobileNet

Configuration	1	2	3	4	5	6	7	8	9
Number of epochs	50	50	50	50	150	150	150	150	150
Solver type	SGD	SGD	Adam	Adam	Adam	Adam	Adam	Adam	Adam
Learning rate policy	Step	Reduce on plateau	Step	Reduce on plateau	Step	Reduce on plateau	Step	Reduce on plateau	Reduce on plateau
Drop out	0.5	0.5	0.5	0.5	0.5	0.5	0.8	0.8	0.2
Quantized accuracy, %	96.5	96.0	98.62	96.5	97.88	97.62	97.88	99	98.75

In a subsequent experiment with MobileNetV2, the configurations were narrowed down to the top three that demonstrated the best performance with FD_MobileNet. The goal was to identify the optimal setup tailored specifically for MobileNetV2, as outlined in Table 5.

**Table 5:** Experiment 2 parameter selection for MobileNetV2

Configuration	1	2	3
Number of epochs	50	150	150
Solver type	Adam	Adam	Adam
Learning rate policy	Step	Reduce on plateau	Reduce on plateau
Drop out	0.5	0.8	0.2
Quantized accuracy, %	98.88	99	98.5

The training results suggest that the optimal hyperparameter configuration for MobileNetV2 closely mirrors that of FD_MobileNet. This alignment in performance is largely due to the shared architectural foundations between the two models, with FD_MobileNet being a streamlined version derived from the original MobileNet design. The similarities in their core structures—such as the use of depthwise separable convolutions—allow both models to benefit from similar optimization strategies. These findings highlight the robustness of the MobileNet family’s architectural principles, which not only enable effective performance across different versions but also simplify the tuning of hyperparameters, ensuring that key optimizations apply consistently across various model variations. This architectural cohesion ultimately supports the efficient balance between accuracy and computational resource management, making both models suitable for deployment in resource-constrained environments.

After identifying the optimal hyperparameter configurations for FD_MobileNet and MobileNetV2, the models were trained using a combined dataset of 10 classes. This training adopted a balanced dataset enhanced through double augmentation; a method selected for its previously demonstrated high accuracy. The goal was to further improve model performance by capitalizing on the benefits of double augmentation.

As shown in Table 6, it was found that FD_MobileNet generally achieves higher accuracy, particularly with the larger dataset of 10 classes. Both models show comparable accuracy when trained on a smaller dataset of four classes. However, FD_MobileNet demonstrates superior performance with the larger, combined dataset. This difference is notable even though both models were configured with the same *alpha* (width multiplier) of 0.5, maintaining consistent information capacity across different dataset sizes. This consistency highlights FD_MobileNet's effectiveness due to its fast down sampling strategy, which improves performance within the constraints of limited information capacity, crucial for maintaining accuracy in compact models. MobileNet V2, with its complex and deep architecture, is designed to capture more detailed features but incurs higher computational demands. This design choice leads to longer inference times, and greater RAM and flash storage needs compared to FD_MobileNet. In contrast, FD_MobileNet utilizes a strategic fast down sampling approach, reducing spatial dimensions by 32 × in just 12 layers—half the layers required by its predecessor. This efficient method significantly reduces computational costs, enhances the model’s capacity to manage and process information without enlarging its size, and optimizes memory and cache usage, thereby minimizing switches and boosting efficiency. These advantages underscore FD_MobileNet’s ability to balance compact model dimensions with strong performance, particularly valuable in resource-limited applications.

**Table 6:** Performance of FD_Mobilenet and MobileNetV2

Model type	FD_MobileNet	MobileNetV2
Accuracy, %	95.79	94.57
Inference time, ms	134.6	289.8
Flash size, KiB	550	525
Ram size, KiB	206.88	717

### Model deployment – evaluation model performance on microcontroller

Both FD_MobileNet and MobileNetV2 are deployed on the STM32H747i board to evaluate hardware performance. To standardize the evaluation, 30 images from each class were randomly selected from the test dataset, distinct from the training set to ensure unbiased evaluation. These images were displayed on a screen to assess the model's accuracy.

**Fig. 8:**
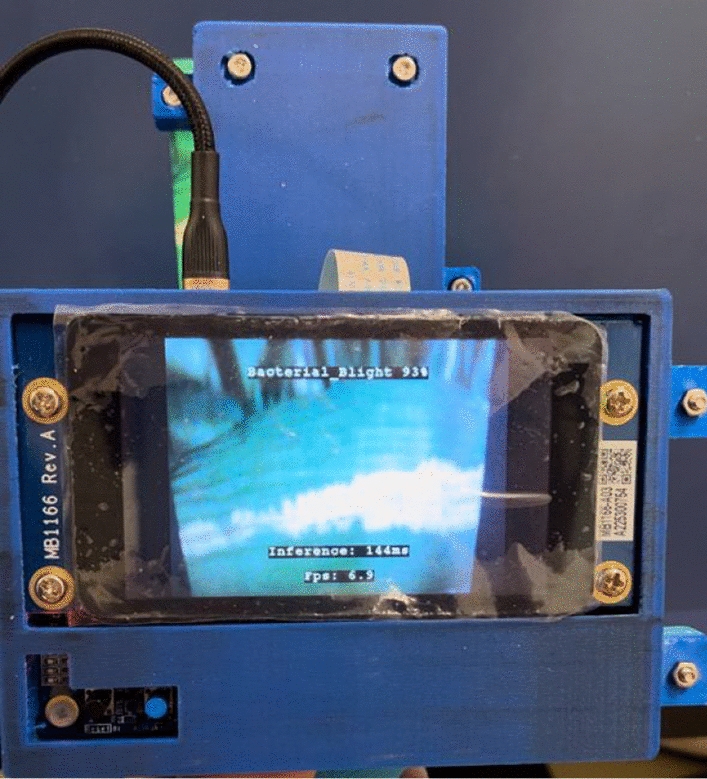
Evaluation of performance of model after deployment

The evaluation aimed to replicate real-world conditions likely to be encountered in paddy fields, where the device would typically be handheld as illustrated in Fig. 8. This meant that the camera's distance and angle were not fixed during the tests, simulating the variable conditions under which users might operate the device. To validate the model’s accuracy under these conditions, five volunteers were recruited to use the device and collect data. The average accuracy from these trials was calculated, providing a measure of the model’s practical performance.

Deploying the MobileNetV2 model, trained on a balanced double augmentation dataset encompassing 10 classes, onto the STM32H747i board, revealed a performance bottleneck akin to that observed with FD_MobileNet. The STM32H747i board is equipped with 1 MB of RAM and 2 MB of flash memory, but a significant portion—approximately 70% of RAM and 50% of the flash—is allocated to the AI model. This leaves only a limited number of resources, specifically over 300 KB of RAM and more than 1000 KB of flash, for other essential functions such as camera operation, image processing, and LCD display management.

This allocation of resources implies that while the model might perform adequately with a smaller number of classes, which require less intensive image processing, the challenges increase as the number of classes grows. The detailed processing necessary for a higher number of classes struggles to receive adequate support from the limited remaining resources. This discrepancy in model performance between hardware deployment and simulation stages can be attributed to the vast difference in processing capabilities. In simulations, image processing benefits from the substantial power of a 6 GB RTX GEFORCE 3060 graphics card and a 12th Gen Intel i9 core, which far exceeds the computational resources available on the STM32H747i board. To investigate the integrity of the deployment process and the accuracy of camera and image processing settings, a model trained with a simpler 4-class dataset was deployed. Table 7 shows the result from the deployment of the FD_MobileNet.

**Table 7:** Evaluation result of FD_MobileNet with 4 classes

Class	Number of false positive
Bacterial blight	1
Blast	1
Brown spot	3
Tungro	7
Total	12

From these results, the model demonstrated an accuracy rate of 90% across 120 images. While this performance is commendable, it falls short of the simulation accuracy of 99%. Various factors inherent to the real-world deployment environment contributed to this discrepancy, including lighting conditions, camera distance, and angle variability. Unlike in simulations where test images are fed directly into the model, hardware deployment introduces variables such as screen brightness and ambient lighting, which can impact image capture. Additionally, the variability in user handling, with six different users operating the device without specific instructions on zooming or angling, reflects the diverse conditions expected in practical settings.

Notably, the model showed high effectiveness in recognizing bacterial blight and blast, with accuracies approaching 97%, suggesting that it captures these conditions well despite environmental variables. However, the performance for brown spot and tungro diseases was lower, with a significant number of false positives, potentially indicating the model's sensitivity to the environmental factors.

Table 8 shows the evaluation of MobilNetV2 model. The results indicate that MobileNetV2 exhibits superior performance with a 97.5% accuracy rate, accurately identifying bacterial blight and blast diseases. It shows a distinct advantage over FD_MobileNet, particularly in detecting Tungro, where MobileNetV2 achieves a 93% accuracy, significantly outperforming FD_MobileNet's 77%. This difference highlights the architectural distinctions between the two models. MobileNetV2, with its complex structure, excels at identifying subtle features but requires more computational resources. In contrast, FD_MobileNet employs a fast-down sampling strategy that rapidly reduces spatial dimensions by 32 × within just the first 12 layers—half the number used by MobileNet. This approach allows FD_MobileNet to effectively recognize diseases with clear visual markers, such as bacterial blight, blast, and brown spot disease.

**Table 8:** Evaluation result of MobileNetV2 with 4 classes

Class	Number of false positive
Bacterial blight	0
Blast	0
Brown spot	1
Tungro	2
Total	3

However, FD_MobileNet faces challenges in detecting Tungro, a disease that lacks distinctive visual cues in its training images, making it difficult to differentiate from other conditions based solely on the appearance of green leaves. This difficulty in identifying Tungro with FD_MobileNet, but not in simulation results, underscores the influence of environmental factors during hardware evaluations. While simulations provide direct image input to the model, ensuring clarity and accuracy, hardware tests are subject to external variables that introduce complexities absent in controlled environments.

To further explore the impact of resource constraints on model performance, two experiments were designed. The first experiment tests the application of MobileNetV2, starting with 4 classes and incrementally increasing the number. This experiment aims to determine the model’s performance threshold in relation to the number of classes and to pinpoint when performance begins to degrade significantly. The second experiment focuses on resource optimization for image processing. By reallocating more resources to image processing and accepting a longer inference time as a trade-off, this experiment intends to assess whether improved image processing capabilities can enhance model performance on the hardware. These experiments are designed to systematically investigate and address the challenges faced by deep learning models when deployed in resource-limited environments, such as the STM32H747i board.

**Fig. 9:**
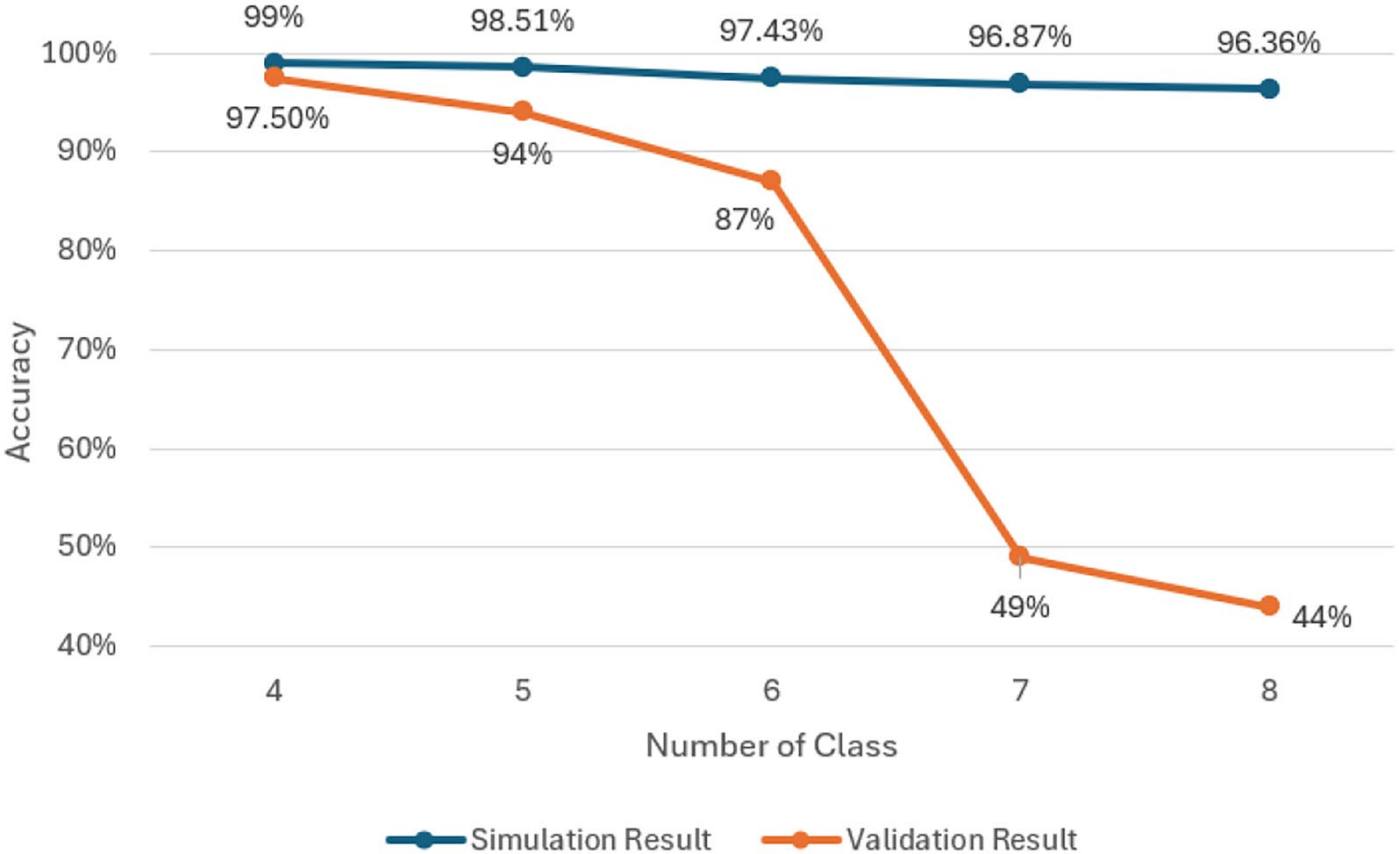
Comparison of simulation result and validation result for variable class sizes

Table 9 and Fig. 9 illustrate the impact of varying class sizes on model accuracy in both simulation and hardware validation. The results demonstrate a clear trend of declining accuracy as the number of classes increases, which is an expected outcome given the fixed computational resources allocated by the width multiplier. Since the width multiplier remains constant throughout the experiments, the available computational resources must be shared among a growing number of classes. This resource distribution results in a gradual decline in model performance, as more classes introduce additional complexity for the model to process and classify accurately.

**Table 9:** Simulation and validation result for variable class sizes

Number of classes	Simulation result (%)	Validation result (%)
4	99	97.5
5	98.51	94
6	97.43	87
7	96.87	49
8	96.36	44

In the hardware validation phase, this downward trend in accuracy becomes more pronounced, highlighting the constraints imposed by the STM32H747i board. The model achieves an accuracy above 90% when tested with up to five classes, indicating that the hardware can efficiently handle a moderate level of classification complexity. However, once the number of classes increases to six, the accuracy drops significantly to 87%, representing a 7% decrease. This decline suggests that the model is starting to stretch the hardware's computational capabilities as more classes are introduced.

When the number of classes is further extended to seven, the accuracy plummets to 49%, signalling a substantial performance bottleneck. This drastic reduction in accuracy implies that the STM32H747i board is reaching its operational limits, as the model struggles to allocate sufficient resources to accurately classify the growing number of classes. The sudden drop in performance highlights the challenges of deploying deep learning models in resource-constrained environments, particularly when handling more complex classification tasks.

This pattern of diminishing returns as the class size increases emphasizes the importance of optimizing the balance between model complexity and hardware capabilities. Without careful resource management, increasing the number of classes can significantly degrade performance, particularly in embedded systems with limited computational power, such as the STM32H747i microcontroller. This underscores the need for targeted optimization strategies, such as model compression and resource allocation adjustments, to enhance performance under hardware limitations.

Table 10 shows the impact of various model optimization strategies on the resource utilization and accuracy of MobileNetV2 when implemented on the STM32H747i-Disco board. The initial optimization strategy adopted a balanced approach, focusing on both RAM size and inference time. Subsequently, the focus shifted to primarily reducing RAM usage, with the goal of decreasing the resources required by the model. Following this targeted optimization for RAM size, there was a slight reduction in RAM consumption by approximately 0.5%, and a decrease in flash size by about 1.14%. Despite these relatively minor changes in resource usage, there was a significant improvement in validation accuracy of 9%. This outcome suggests that even modest optimizations in computational resource management can have a substantial effect on model performance.

**Table 10:** Result of resources optimization

Optimization option	Balance	Optimize RAM
Flash size, KB	525	519
RAM size, KB	713	713
Inference time, ms	289.8	377.8
Validation accuracy, %	49%	58%

The improvement in accuracy following optimization was notably apparent in the model’s ability to recognize Bacterial Leaf Streak. Before optimization, the model was unable to correctly identify any images from this class. After optimization, there was a significant enhancement, with the model correctly identifying over 50% of Bacterial Leaf Streak images. However, the accuracy for Tungro and Normal classes did not exhibit similar improvements, indicating that the image processing capabilities available might still be insufficient for the model to effectively distinguish these classes.

Given that the RAM and flash sizes are already at their operational limits for running the model, it is evident that hardware constraints play a significant role. The minor optimization in RAM usage, which led to longer inference times, appears to provide a trade-off that could be advantageous for accuracy in certain classes. This observation lays a foundation for further investigation into the balance between computational resources and model performance, especially for complex tasks like image classification on edge devices.

## Conclusion

The deployment of AI, specifically CNNs, for rice disease detection using ARM-M microcontrollers, shows great promise in transforming agriculture, particularly in regions like Malaysia, where rice is a staple. AI technologies, such as MobileNetV2 and FD-MobileNet, demonstrated significant improvements in early disease detection, with MobileNet V2 achieving 97.5% accuracy and FD-MobileNet reaching 90% under controlled conditions. These improvements can help reduce crop losses and enhance productivity.

However, real-world deployment presents challenges due to hardware limitations. On the STM32H747i board, equipped with 1MB of RAM and 2MB of flash memory, both models experienced performance bottlenecks. FD-MobileNet’s accuracy dropped to 90% across 120 images in real-world tests. Resource optimization emerged as crucial for maintaining performance, with a modest 0.5% reduction in RAM usage and a 1.14% decrease in flash size, leading to a 9% increase in MobileNetV2’s validation accuracy.

Adaptability to environmental conditions was also critical. Initially, FD-MobileNet struggled to identify Bacterial Leaf Streak, but after optimization, its accuracy improved to over 50%, highlighting the model’s potential with proper fine-tuning.

In conclusion, while AI presents a powerful tool for enhancing agricultural productivity, its deployment requires careful consideration of hardware constraints and environmental conditions. Continued efforts to optimize AI models, both in terms of accuracy and resource efficiency, will be essential for their successful application in agriculture. These advancements are key to realizing AI's full potential in promoting sustainable and effective farming practices.

However, the application of these technologies in real-world scenarios has encountered several challenges. Key among these is the limitation imposed by the hardware used in the field. For instance, during our experiments, the performance of models like MobileNetV2 and FD-MobileNet varied significantly under different conditions. In controlled environments, these models achieved high accuracy rates—MobileNetV2 reached an accuracy of 97.5%, and FD-MobileNet showed a slight underperformance in comparison. Yet, when deployed on STM32H747i boards, which have just 1MB of RAM and 2MB of flash memory, the models encountered performance bottlenecks. Specifically, the accuracy of FD-MobileNet dipped to 90% across 120 images when tested under varied real-world conditions.

Resource optimization emerged as a crucial factor in maintaining model efficacy. Small improvements in computational resource management significantly boosted model performance. For instance, a modest optimization in RAM usage resulted in an appreciable 9% increase in validation accuracy for MobileNetV2, indicating that even slight enhancements can yield substantial benefits.

Moreover, the adaptability of models to different environmental conditions and disease manifestations without extensive manual recalibrations proved essential. For example, while the FD-MobileNet model was initially unable to correctly identify any images of the Bacterial Leaf Streak class, after optimization, it correctly identified over 50% of these images, showcasing a significant improvement.

In summary, while AI presents a promising solution to many of the challenges faced in agricultural disease detection, the actual deployment of these technologies in the field requires careful consideration of hardware capabilities and environmental conditions. Future efforts should focus on developing AI models that are not only accurate but also robust and efficient enough to operate within the constraints of the hardware typically available in agricultural settings. Continued refinement of these models will be key to their successful application, ensuring they can perform reliably in diverse and challenging environments. This research underscores the potential of AI to significantly enhance agricultural productivity and sustainability, provided that ongoing advancements continue to address the practical limitations of technology deployment in the field.

## Data Availability

The supporting data for this study’s findings are not publicly accessible due to intellectual property considerations. Interested parties can request access from the corresponding author.
